# Ultra‐Efficient Photocatalytic Properties in Porous Tungsten Oxide/Graphene Film under Visible Light Irradiation

**DOI:** 10.1002/advs.201500116

**Published:** 2015-06-30

**Authors:** Lin Mei, Haitao Zhao, Bingan Lu

**Affiliations:** ^1^ Key Laboratory for Micro‐Nano Optoelectronic Devices of Ministry of EducationSchool of Physics and ElectronicsHunan UniversityChangsha410082P.R. China; ^2^ Department of Chemistry and BiochemistryUniversity of CaliforniaLos AngelesCA90095USA; ^3^ School of Physical Science and TechnologyLanzhou UniversityLanzhouGansu730000P.R. China; ^4^ Department of ChemistryStanford UniversityStanfordCA94305USA

**Keywords:** graphene, photocatalysis, porous structure, tungsten oxide, visible‐light

## Abstract

Recently, a growing amount of effort has been devoted to solving the widespread problem of pollution. Photocatalysts have attracted increasing attention for their widespread environmental applications. Here, a classic and simple electrospun technique is used to directly fabricate a porous a tungsten oxide nanoframework with graphene film as a photocatalyst for degradation of pollutants. The as‐synthesized film simultaneously possesses substantial adsorptivity of aromatic molecules, extensive light absorption range, significant light trapping, and efficient charge carrier separation properties, which remarkably enhance photocatalytic activity. In the photodegradation of Rhodamine B, a significant photocatalytic enhancement in the reaction rate is observed, which has superior photocatalytic activity compared to other bare WO_3_ and TiO_2_ nanomaterials under visible‐light irradiation.

## 1 Introduction

For the sustainable development of human society, the development of both pollution‐free technologies for environmental remediation and alternative clean energy supplies is an urgent task.^1^ To date, among the wide variety of green earth and renewable energy projects underway, semiconductor photo­catalysis has emerged as one of the most promising technologies because it represents an easy way to utilize the energy of either natural sunlight or artificial indoor illumination, and is thus abundantly available everywhere in the world.^1^, ^2^, ^3^, ^4^ TiO_2_ is a promising candidate, but its band gap (*E*_g_ ≈ 3.2 eV) limits the photocatalytic activity of TiO_2_ to ultraviolet range.^5^, ^6^, ^7^, ^8^ In contrast, WO_3_ with a narrower band gap (e.g., *E*_g_ ≈ 2.6 eV) provides the opportunity to harvest visible light.^9^, ^10^ Nevertheless, pure WO_3_ are usually not efficient photocatalysts, due to its relatively low conduction band level (0.5 V vs NHE, normal hydrogen electrode), high electron‐hole recombination rate and difficulty in the reduction of oxygen.^11^ Although progress has been made to improve the behavior of WO_3_,^12^, ^13^, ^14^, ^15^ several problems still hinder further promotion of efficiency of the present WO_3_ composites, such as the marked decrease of the adsorptivity during photodegradation, the weakening of the light intensity arriving at catalysts' surface, and the high electron‐hole recombination rate, etc. In this regard, it is still an imperative and challenging issue to develop new and ultra‐efficient visible‐light‐sensitive photocatalysts base on WO_3_.

In previous research, nanomaterials using as photocatalysts are powder, which resulted in improved photocatalytic activity (**Table** **1**). Thus far, there is no reported preparation of graphene nano‐framework as films with high performance of photocatalytic active. Graphene possesses unique physicochemical properties including large surface area, good flexibility, high electrical conductivity, high chemical stability, and the high transparency.^22^, ^23^ It is important that graphene served as an acceptor of the photogenerated charge carriers which can prevent the recombination of electron‐hole pairs. Thus, the combination of photocatalysts and graphene is promising to simultaneously possess excellent adsorptivity, transparency, conductivity, and controllability, which could facilitate effective photodegradation of pollutants.^24^, ^25^ However, graphene is so flexible that it is difficult to use directly, usually needing a support, such as being coated on the surface of nanofibers or doping in nanofibers.^26^ Yet in the practical application, coating only on the surface is easy to fall off. And doping graphene in nanofibers cannot completely show the superiority. So, for practical application, it is very important to construct exposed graphene and not easily fall off.

**Table 1:** Photocatalytic properties of advanced photocatalysts

Photocatalyst	Mass fraction of compound	Photocatalytic experiments	Photocatalytic activity	Refs.
N‐doping HNb_3_O_8_	–	Decomposing RhB under visible‐light irradiation	Complete degradation (CP) in 50 min	16
Ag/AgCl	8/92	Decomposing MO under visible‐light irradiation	CP in 8 min	17
BiVO_4_	–	Decomposing RhB under visible‐light irradiation	CP in 30 min	18
Ag_3_PO_4_ rhombic dodecahedrons	–	Decomposing MO under visible‐light irradiation	CP in 4 min	19
Fe_3_O_4_/WO_3_ core–shell microspheres	No data	Decomposing MB under visible‐light irradiation	CP in 90 min	14
		Decomposing RhB under visible‐light irradiation	CP in 120 min	
Black TiO_2_	–	Decomposing MB under visible‐light irradiation	CP in 8 min	6
Carbon‐Coated CdS	1.0 g glucose	Decomposing MO under visible‐light irradiation	Degradation percentage (DP) of 96.6% in 40 min	20
Bi_2_WO_6_–G	No data	Decomposing RhB under visible‐light irradiation	DP of 90% in 4 min	15
InNbO_4_–G	No data	Decomposing MB under visible‐light irradiation	DP of 97.6% in 90 min	21
TiO_2_–G	G: 0.6 wt%	Decomposing MB under UV light	Reaction rate: 0.071 min^−1^	7
TiO_2_–GO	No data	Decomposing MB under UV light	DP of 99% in 15 min	8

In response to this call, we developed a simple, electrospun technique for synthesizing porous tungsten oxide nano‐framework with graphene film (GWF). As expected, graphene film can be exposed in the porous WO_3_ nano‐framework, and which can completely show the superiority of the graphene. The GWF showed superior performance in the photocatalytic properties under visible light irradiation (irradiation of 6 min, degradation rate can arrive 81.8%, and only 15 min irradiation, almost 100% of the RhB molecules were decomposed). The reason for the high photocatalytic activity was also investigated.

## 2 Results and Discussion

**Figure 1:**
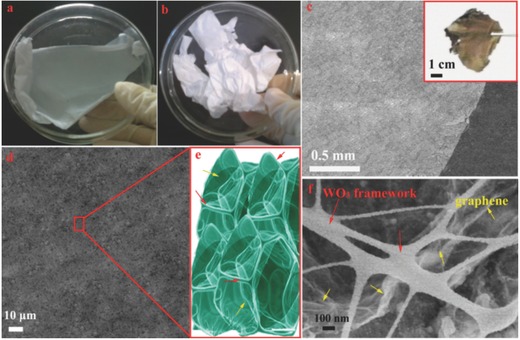
Photographs of a) as spun PVP/WCl_6_/graphene film b) as PVP/WCl_6_ film; c,d) Low‐magnification SEM image of GWF (inset is the photograph of GWF); e) Structure chart of GWF; and f) High‐magnification SEM image of GWF.

Graphene was introduced into WCl_6_ and polyvinyl pyrrolidone (PVP) solution as the precursor for electrospun. After annealing in air, the final porous nanoframework film was formed, which evolve from the PVP molecules volatilized. **Figure** **1**a,b shows the precursor PVP/WCl_6_/graphene and PVP/WCl_6_ respectively, which as prepared by the electrospun technique. It is apparent from Figure 1a,b that the color of as‐obtained film with and without graphene are light gray and pure white, respectively, which is an indirect proof of the homodisperse nature of graphene in as‐spun nanofibers. Before annealing, the precursor was uniform fibers with smooth surface (Figure S1a, Supporting Information). After annealing in air, the hybrid materials formed a film structure (Figure 1c,d). From the Figure 1f, it is clear that the WO_3_ nanofibers in hybrid materials can be used as the nanoframework to support the integrality of architecture and act as pillars (made graphene not easily fall off) and effectively separate graphene nanosheets. Specially, plentiful graphene nanosheets can be exposed in the GWF resulting from the WO_3_ nanofibers as the nanoframework. Compared with GWF, we synthesized the WO_3_ nanofibers without graphene nanosheets as shown in Figure S1 (Supporting Information). The morphology of the pure WO_3_ is uniform nanofibers with a diameter of around 100 nm.

**Figure 2:**
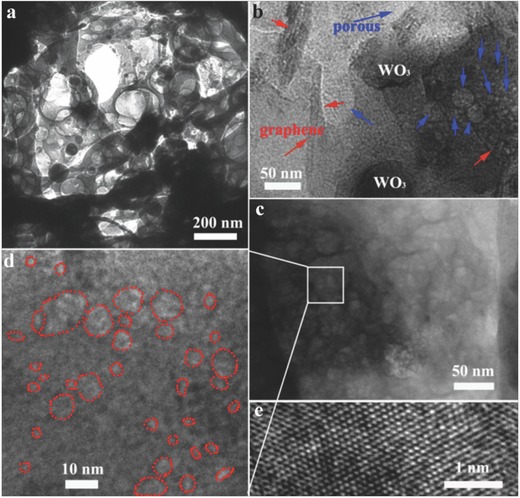
a–d) TEM images of the GWF, e) HRTEM image of the GWF. The red arrows: graphene and blue arrows: porous structures.

It is interesting to see from the transmission electronic micrograph (TEM) image (**Figure** **2**a) of an inner film structure that the microstructure consists of remarkable unordered pores and cotton‐like nanofibers. The WO_3_ nanofibers increase the number of micro‐ and mesopores to enhance the specific surface area. It is not obviously observe the graphene nanosheets in this TEM magnification. Figure 2b TEM image can directly demonstrate the homodisperse nature of graphene nanosheets in film structure. The pore‐size characterization of the GWF and bare WO_3_ was further verified by measuring the nitrogen adsorption/desorption isotherms (Figure S2a, Supporting Information). The GWF samples have a higher surface area comparing with the bare WO_3_. Typically, the Brunauer–Emmett–Teller (BET) surface area of GWF was 56.4 m^2^ g^−1^, total pore volume was 0.69 cm^3^ g^−1^, mesopore volume was 0.56 cm^3^ g^−1^, mesopore to total pore‐volume ratio was 0.81, and an average pore diameter was 8.2 nm (Figure S2b, Supporting Information). The surface area of GWF is higher than pure WO_3_ nanofibers result of graphene nanosheets and porous structure. The responding density functional theory (DFT) pore size distribution (Figure S3, Supporting Information) exhibits a hierarchical pore structure that includes micro‐, meso‐, and macropores (>50 nm). The results are consistent with that of the TEM data. The pore size distribution of GWF samples is wide, indicating unordered mesopores. Clearly, the original unordered mesopores maintain their structure by heat treatment. The most remarkable features of the as‐synthesized GWF are the high volume of mesopores and the high specific surface area, as well as the high photocatalytic activity under visible light. To understand the growth mechanism and substantial factors of porous film, precursor were annealing at different temperatures (350–550°C) and their scanning electron microscopy (SEM) images and growth mechanism have been given in Figure S4 (Supporting Information). In the precursor, the graphene nanosheets were curled up in the nanofibers. When annealing at high temperature, the curled graphene nanosheets can be extended with the PVP molecular decomposite. Meanwhile, the WO_3_ nanofibers form stable the framework on the surface of graphene nanosheets. When the annealing temperature is lower 350 °C, the PVP molecular cannot be decomposite completely. As the annealing temperature is higher than 450 °C in air, the graphene nanosheets can be oxidized, as shown in Figure S4 (Supporting Information).

**Figure 3:**
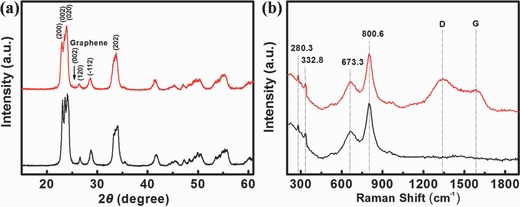
a) The XRD patterns and b) Raman spectra of porous GWF (red line), bare WO_3_ (black line).

Figure 2e shows the high‐resolution TEM images of GWF, from which we determined the interplanar distances to be 0.26 nm, which corresponds well to the lattice spacing of the [202] plane of WO_3_. The crystal structure of the WO_3_ and GWF was further characterized by X‐ray diffraction (XRD), as shown in **Figure** **3**a. The diffraction peaks corresponding to the monoclinic WO_3_ [200], [002], [020] and [202] planes (JCPDS no. 71‐0131) were detected along with the characteristic features of graphene. In addition, it is clear observed that the [002] in GWF was the characteristic peak of graphene. From the XRD patterns of bare WO3, no peaks associated with other compounds were detected. The GWF was calculated from the TGA in air (Figure S5, Supporting Information). The sample showed a significant weight loss between 520 °C and 620 °C due to the decomposition of graphene, and the composite contains ≈10% graphene. Meanwhile, we fabricated different graphene concentrations from 5% to 15% in mass ratio in WO_3_ nanoframework structure. We found the higher and lower graphene concentration in hybrid materials cannot be form the stable porous film.

The Raman scattering spectrum also confirms the formation of the GWF, showing the two strongest peaks at 673.3 and 800.6 cm^−1^ assigned to the stretching vibration of tungsten atom with neighboring oxygen atoms [υ (OWO)] (Figure 3b). The peaks at around 280.3 cm^−1^ accounts for the bending vibration [δ (OWO)].^13^ Concerning the carbonaceous materials, the well‐known characteristics of Raman spectra are the D and G bands (≈1350 and 1585 cm^−1^), which are assigned to the local defects/disorders and the *sp^2^* graphitized structure, respectively. This means that greater *I*_D_/*I*_G_ peak intensity ratio can be attributed to higher defects/disorders in a graphitized structure such as graphene. From the shape of peaks at ≈1350 and 1585 cm^−1^, it is determined that the graphene is not exposed to the surface of the film.^27^, ^28^, ^29^

**Figure 4:**
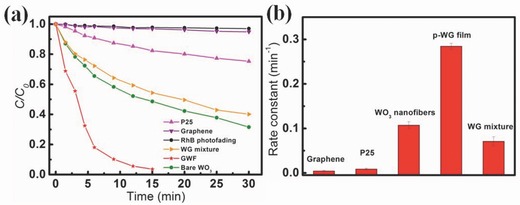
a) Comparison of photocatalytic degradation rates of RhB, and b) Average reaction rate constant (min^−1^) for the photodegradation of RhB. The error bars are based on measurements on at least three different samples.

The photocatalytic activities of GWF (50 mg) were evaluated using the photodegradation of Rhodamine B (RhB, initial concentration 20 mg L^−1^; 50 mL) under visible‐light irradiation as a probe reaction, and the result is shown in **Figure** **4**a. The value of (*C*/*C*_0_), the instantaneous concentration of RhB during the photodegradation normalized to the initial concentration *C*_0_, was proportional to the normalized maximum absorbance (*A*/*A*_0_), which was measured at *λ* = 554 nm at regular time intervals. It can be seen that only after 3 min irradiation 44.5%, 21.8%, and 4.6% of the initial dye was decomposed by GWF, bare WO_3_ nanofibers, and P25 as the photocatalyst, respectively. After 6 min of irradiation, only 18.2% of the initial dye remained in the solution for GWF used as the photocatalyst (Figure S6, Supporting Information). In contrast, nearly 65.5% and 90.8% of the initial dye still remained for bare WO_3_ nanofibers and P25 used as the photocatalyst after 6 min irradiation. It could be observed that GWF framework structures exhibited more prominent visible‐light photocatalytic activity. Continued irradiation, after total 15 min irradiation, almost 100% of the RhB molecules were decomposed, but 48.6 and 82.3% RhB molecules were intact when bare WO_3_ nanofibers and P25 were used as the photocatalyst (Figure 4a). The physical mixture of WO_3_ and graphene (WG mixture) showed poorer activity in the photodegradation compared to the GWF (72.2% and 40.2% of the initial dye remained after irradiation of 6 and 30 min). Accordingly, the exposed graphene plays an important role in the photodegradation, and the adsorption of RhB molecules contributed to the enhanced photoactivity.

The kinetics of the degradation reaction were fitted to a pseudo‐first‐order rate law at low dye concentration using the equation ln (*C*_0_/*C*) = *kt*, where *k* is the apparent rate constant and *t* is the irradiation time.^30^ From Figure 4b, one can see that the average values of *k* for GWF and bare WO_3_ were 0.2846 and 0.071 min^−1^, respectively. Furthermore, we note that the activity of GWF was ≈32 times those of P25 (*k* = 0.0089 min^−1^) and higher than those of other reported TiO_2_ and TiO_2_/graphene composites.^7^, ^8^, ^23^, ^24^, ^25^ In addition to efficiency, stability and recyclability of photocatalysts are also important features for applications. After the RhB molecules are completely decomposed, centrifugation of the solution enables the GWF to be collected to catalyze a new reaction. Figure S7 (Supporting Information) plots curves for degradation of RhB solution under the same experimental conditions. The GWF photocatalyst can be effectively recycled at least six times without any obvious decrease the removal rate of RhB, which demonstrates their high stability. From Figure S8 (Supporting Information) (SEM image of GWF photocatalyst after six times recycled) can know the morphology of GWF almost no change. In addition, this is presumably because of the photocatalytic process. The catalyst change is only a physical process as the structure of the catalyst is not influenced.^5^

**Figure 5:**
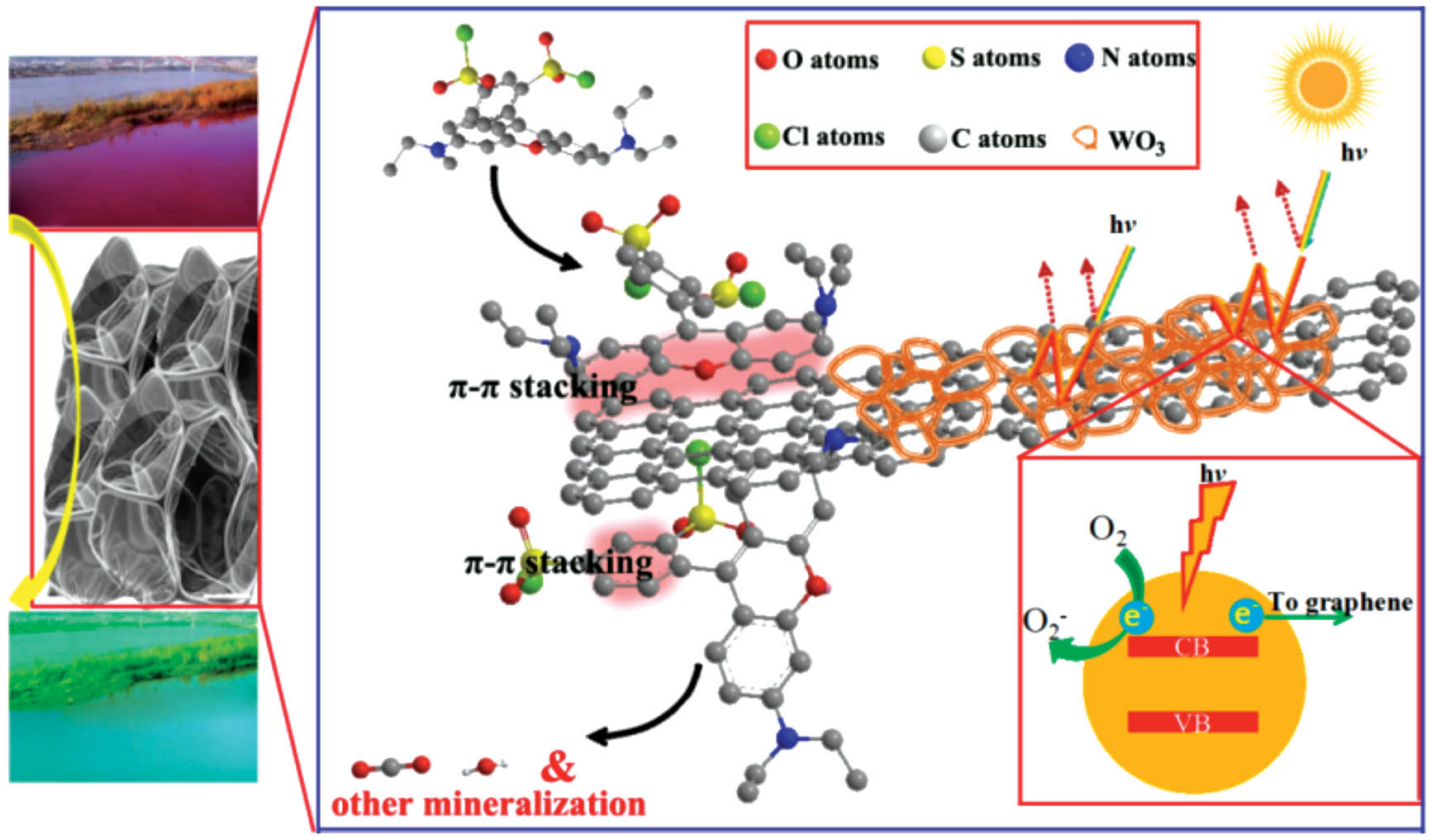
Schematic illustration of processes of the photodegradation of RhB on the porous GWF.

We proposed **Figure** **5** for the photocatalysis process. First, the WO_3_ nanofibers can be used as the nanoframework to support the integrality of architecture and plentiful graphene nanosheets can be exposed in the GWF. So many RhB molecules were adsorbed on the graphene with offset face‐to‐face orientation via π–π conjugation until adsorption–desorption equilibrium.^31^, ^32^ Under the visible‐light irradiation, the excited electrons and holes migrate to the WO_3_ surface and then the electrons react with exposed graphene nanosheets, which served as an electron acceptor.^25^, ^33^ Meanwhile, the visible‐light will be more effectively utilized through a multi reflection effect of porous structures.^34^, ^35^, ^36^ The transmitted visible light will reflect in the hollow channels, which induces a multiple reflection effect, which further improves the collection efficiency of visible light within the films and, consequently, elevates the degradation rate of organic molecules. As the decomposition of RhB molecules, the adsorption equilibrium broke and more RhB molecules would transfer from solution to the interface and subsequently be decomposed into CO_2_, H_2_O and other mineralization through a series of redox reactions. Therefore, there would be a synergetic effect between adsorptivity and photo­reactivity, resulting in an appreciable improvement in photodegradation of RhB. The reason for the ultra‐efficient photocatalytic properties of GWF is discussed in the following. Enhanced adsorptivity of GWF was different from the traditional photocatalysis, which largely assigned to the great physical adsorption. The GWF with larger specific surface areas can absorb more oxygen means more electrons and holes are available. The porosity can improve the photon application efficiency, i.e., the porosity can improve the efficiency of the photocatalysis. Specifically, the GWF showed better adsorption of RhB than bare WO_3_ mainly due to its giant and entire π‐conjugation system and 2D planar structure, and thereby exhibited faster photodegradation of the RhB. The adsorption was noncovalent and driven by the π–π stacking between RhB and aromatic regions of the exposed graphene.^25^, ^31^ Without the visible‐light source, the RhB solution achieved adsorption equilibrium after 15 min, and more than 70% of the initial RhB molecules still remained in the solution after 1 h (Figure S9, Supporting Information). Meanwhile, graphene is a competitive candidate for the acceptor materials due to its 2D π‐conjugation structure.^37^ In the GWF hybrid films, the photogenerated electrons of WO_3_ could transfer from the conduction band to graphene via a percolation mechanism.^34^ Graphene served as an acceptor of the photogenerated electrons of WO_3_ and effectively suppressed the electron‐hole recombination, promoting the degradation of RhB molecules due to graphene has excellent conductivity. Therefore, the rapid transport of electrons and effective charge separation could be accomplished. As shown in Figure S10 (Supporting Information), the typical electrochemical impedance spectra were presented as Nyquist plots. It is observed that the semicircle of GWF in the plot is shorter than bare WO_3_. This result is indicated a decrease in the solid state interface layer resistance and the charge transfer resistance on the surface of GWF. Overall, both the multiple reflection effect on visible light in porous structures and electron‐accepting and ‐transporting properties of graphene in the composite films could contribute to the considerable improvement in photodegradation.

## 3 Conclusions

In summary, highly porous WO_3_ nanoframework with graphene film photocatalyst with high performance has been successfully and directly produced via the electrospun method. As demonstrated in the present case study, in the GWF, i) plentiful exposed graphene nanosheets can great adsorb RhB molecules via π–π stacking, ii) ultra‐efficient utilization rate of visible‐light irradiation resulting from a multi reflection effect of porous structures, iii) the reduced recombination rate of electrons and holes, and iv) 2D film structure can suitable for adsorption of RhB molecules and charge carriers transportation. A significant photocatalytic enhancement in the reaction rate was observed with GWF, which has superior photocatalytic activity to other bare WO_3_ and TiO_2_ materials under visible light irradiation. This remarkable result is anticipated to open new possibilities in the application of WO_3_ and graphene composites as the photocatalysts in environment remediation. And the preparation methods can be directly used in industrialized production to solve environmental problems occurring nowadays.

## 4 Experimental Section

All solvents and chemicals were of analytical grade, purchased from Beijing Chemical Reagent Factory, and used without further purification.

*Synthesis of Highly Porous Graphene/Tungsten Oxide Nanoframework*: Graphene has been prepared by micromechanical cleavage in which highly oriented pyrolitic graphite is peeled using Scotch tape. In the first step, 0.3 g WCl_6_ (99.9%; Aladdin) was dissolved in 2.3 g ethanol, and this mixed solution was stirred for 30 min. Then 0.003 g graphene was added to above solution and ultrasonic dispersed for 20 min. Second, 0.4 g polyvinylpyrrolidone (PVP) (130 000; Aladdin) was dissolved in 2.2 g *N,N*‐dimethylformamide (DMF) (99.9%; Aladdin) and stirred for 30 min. Then the two prepared solutions were mixed together and stirred for 2 h. Thus, a viscous gel of PVP/graphene/WCl_6_ composite solution was obtained. As for typical electrospun process, the spinneret had an inner diameter of 0.6 mm. Grounded aluminum strips (260 cm in width) with parallel gaps of about 1 cm were used as the collectors. A distance of 15 cm and a direct current voltage of 20 kV were maintained between the tip of the spinneret and the collector. After electrospinning, the fibers were heated from room temperature to 450 °C at a rate of 2.5 °C min^−1^, and then held at 450 °C for 2 h in air.

*Synthesis of Bare WO_3_ Nanofibers*: In the first step, 0.3 g WCl_6_ was dissolved in 2.2 g ethanol, and this mixed solution was stirred for 30 min. Second, 0.4 g PVP was dissolved in 2 g DMF and stirred for 30 min. Then the two prepared solutions were mixed together and stirred for 2 h. Thus, a viscous gel of PVP/WCl6 composite solution was obtained. As for typical electrospinning process, the spinneret had an inner diameter of 0.6 mm. A distance of 15 cm and a direct current voltage of 20 kV were maintained between the tip of the spinneret and the collector. After electrospinning, the fibers were heated from room temperature to 450 °C at a rate of 2.5 °C min^−1^, and then held at 450 °C for 2 h in air.

*Characterization*: The samples were characterized using X‐ray diffraction (XRD, D/Max 2500 with CuK_radiation), scanning electron microscopy (SEM, Hitachi S‐4800), and transmission electronic micrograph (TEM, JEOL‐2010, operated at 200 kV), nitrogen adsorption/desorption (Automated Physisorption and Chemisorption Analyzer, micrometrics, ASAP2020 M+C). The Brunauer‐Emmett‐Teller (BET) method was utilized to calculate the specific surface area using the relative pressure from 0.05 to 0.3. The pore volume (VP) and pore diameter distribution (DP) were derived from the adsorption branches of the isotherms by the Barrett‐Joyner‐Halenda (BJH) model. Thermogravimetric analysis (TGA) were carried out on a DTG‐60H thermogravimetric analyzer with a heating speed of 10 °C/min under air in a flow of 50 mL min^−1^. Raman spectroscopy (JY‐HR800 micro‐Raman). EIS measurements were performed by applying an AC voltage with 5 mV amplitude in a frequency range from 1 to 1 MHz (Chenhua, CHI660b electrochemical workstation).

*Catalytic Activity Testing*: Photocatalytic decomposition of Rhodamine B (RhB) was carried out in a beaker containing a suspension of 50 mg sample in 50 mL RhB solution (20 mg L^−1^) under visible light irradiation. The visible light was taken from xenon lamp with the intensity at wavelength of 420 nm (250 W). During the photodegradation, the temperature was kept at 0 °C. Before the irradiation, the suspensions were kept in the dark for 60 min to ensure the adsorption/desorption equilibrium. At given time intervals (90 s), 3 mL aliquots were sampled and filtered to remove the catalysts. The filtrates were analyzed by recording the variations of the absorption band maximum (554 nm) in the UV–vis spectrum of RhB using a TU‐1901 spectrophotometer.

## Supporting information

As a service to our authors and readers, this journal provides supporting information supplied by the authors. Such materials are peer reviewed and may be re‐organized for online delivery, but are not copy‐edited or typeset. Technical support issues arising from supporting information (other than missing files) should be addressed to the authors.

Supplementary
