# Supplementary material for: Ultra‐Efficient Photocatalytic Properties in Porous Tungsten Oxide/Graphene Film under Visible Light Irradiation
Source: Adv Sci (Weinh). 2015 Jun 30;2(12):1500116. doi: 10.1002/advs.201500116 (PMC5115303; doi:10.1002/advs.201500116)
Supplement: Supplementary file 1 — Supplementary [file ADVS-2-0p-s001.pdf]

Copyright WILEY-VCH Verlag GmbH & Co. KGaA, 69469 Weinheim, Germany, 2015.

ADVANCED SCIENCE
Open Access

Supporting Information

for *Adv. Sci.*, DOI: 10.1002/advs.201500116

Ultra-Efficient Photocatalytic Properties in Porous Tungsten Oxide/Graphene Film under Visible Light Irradiation

*Lin Mei, Haitao Zhao, and Bingan Lu**

Supporting Information

Ultra-efficient Photocatalytic Properties in Porous Tungsten Oxide/Graphene Film under Visible Light Irradiation

*Lin Mei, Haitao Zhao and Bingan Lu**

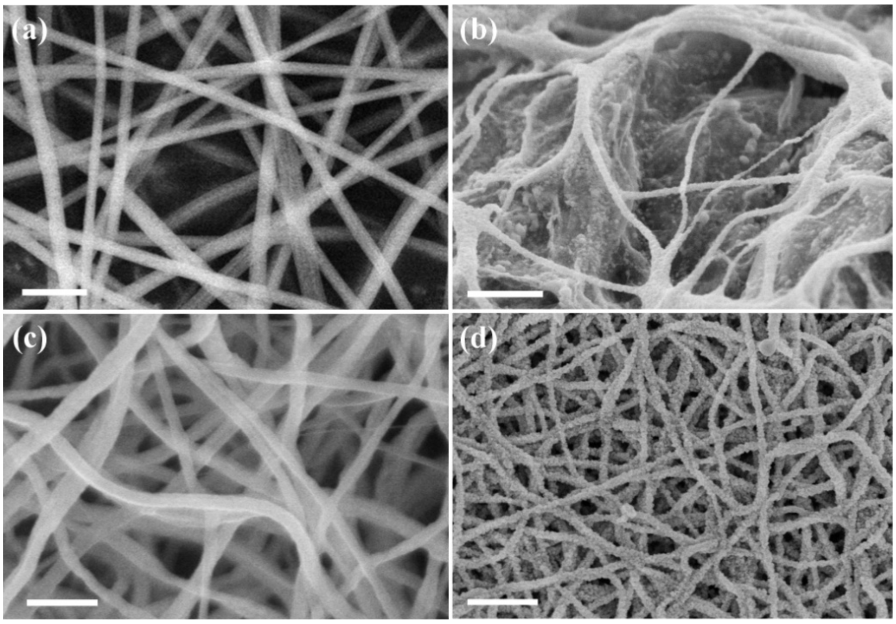

Figure S1. SEM images of (a) the precursor of GWF nanofibers; (b) GWF after annealing; (c) the precursor of WO_3 nanofibers, and (d) WO_3 nanofibers after annealing. The scale bar is 500 nm.

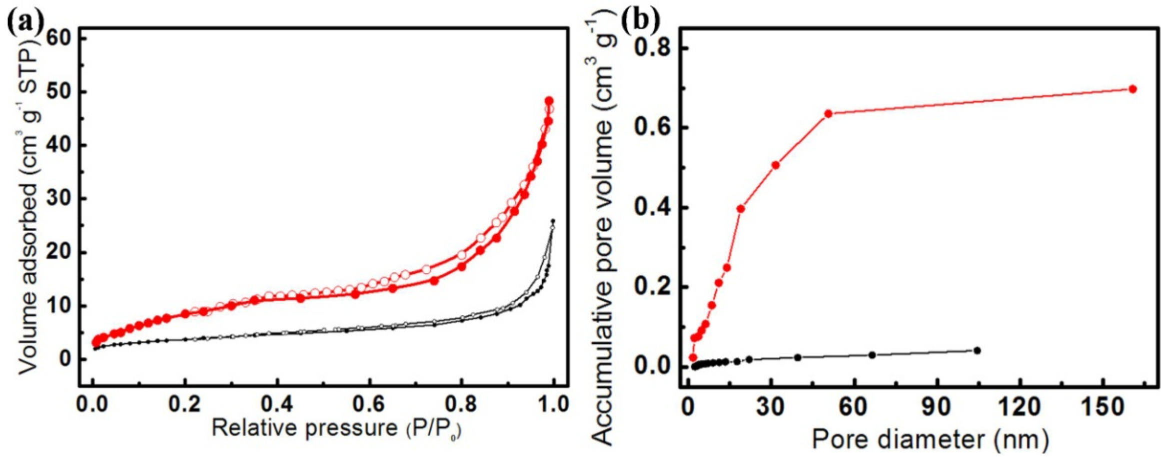

Figure S2. Nitrogen adsorption/desorption isotherms (a) and cumulative pore volume (b) of GWF (red line) and bare WO_3 nanofibers (black line).

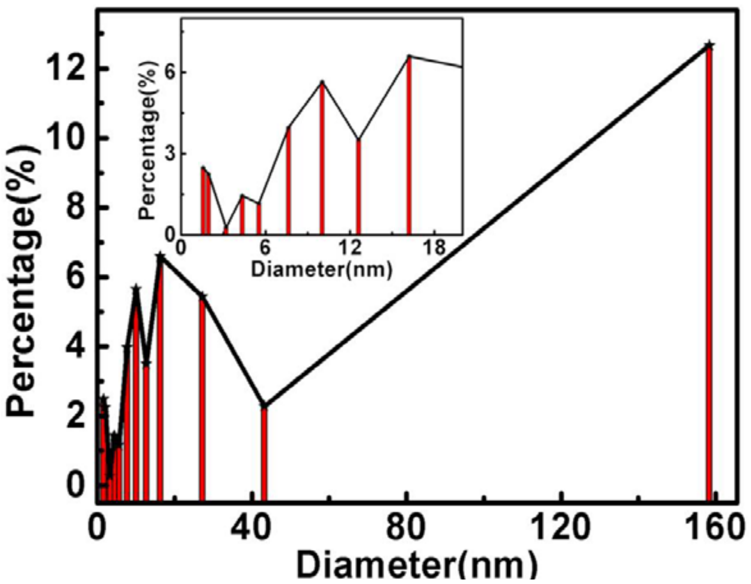

Figure S3. The pore-size distributions of GWF.

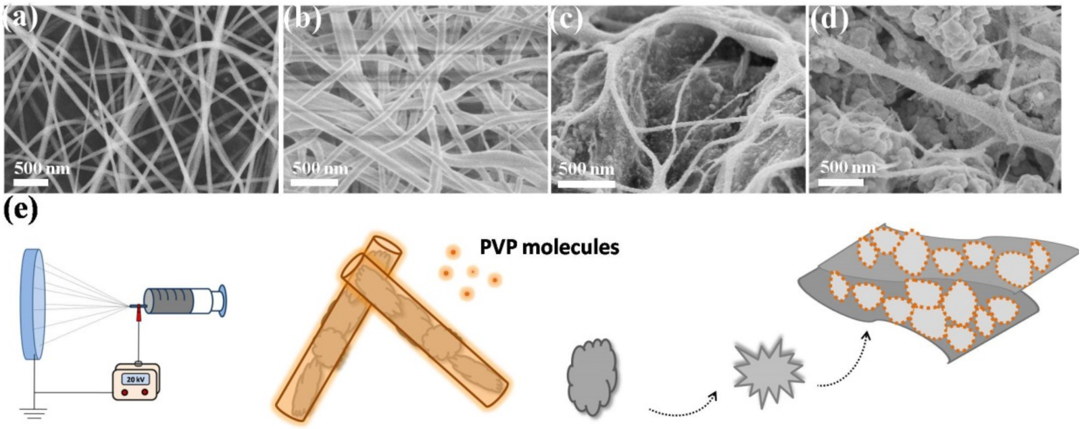

Figure S4. (a-d) SEM images of (a) precursor of GWF, annealing the precursor in different temperature (b) 350 ℃, (c) 450 ℃, and (d) 550 ℃ for 2h. (e) Schematic of growing GWF.

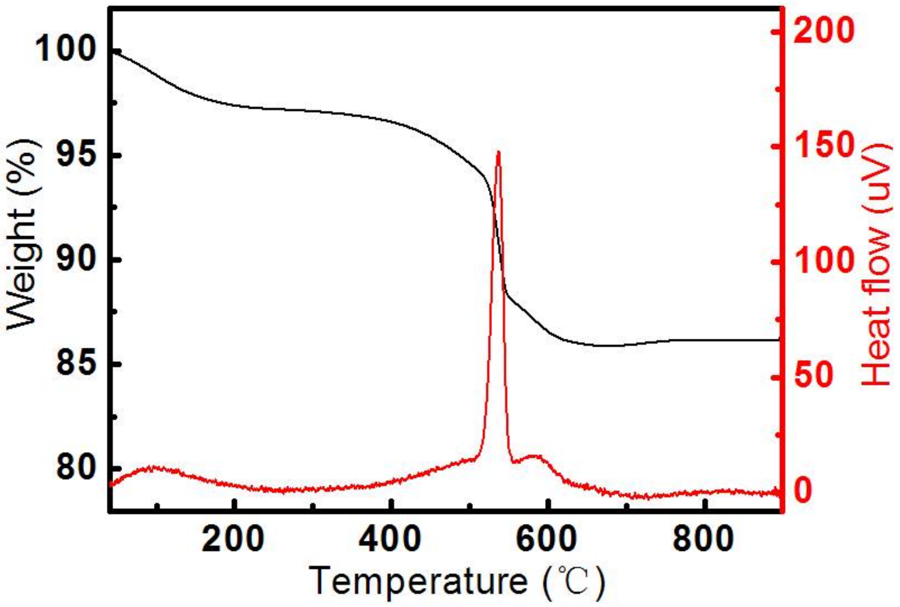

Figure S5. Thermogravimetric analysis curves of GWF.

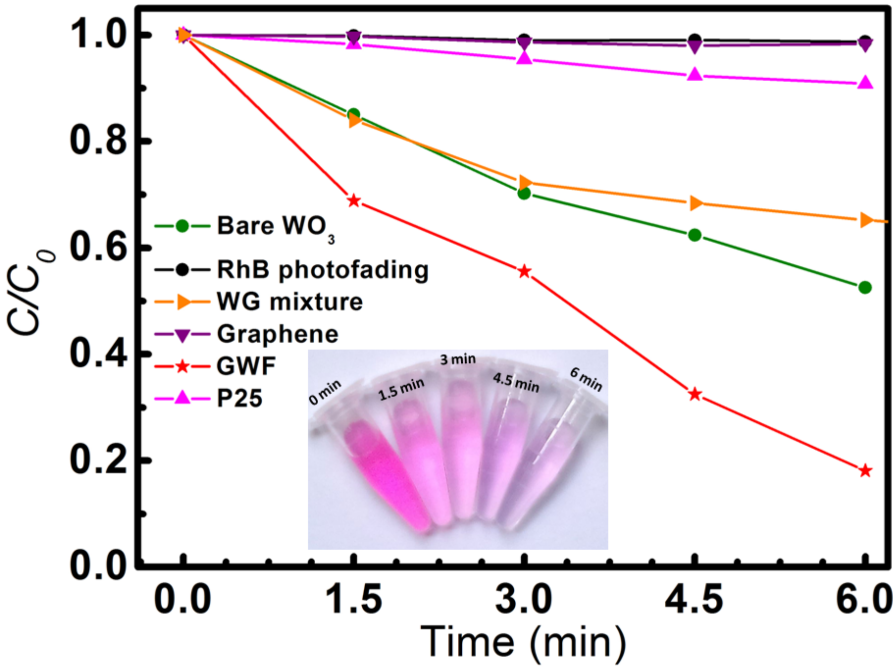

FigureS6. Photocatalytic degradation of RhB monitored as normalized concentration change versus visible-light irradiation time.

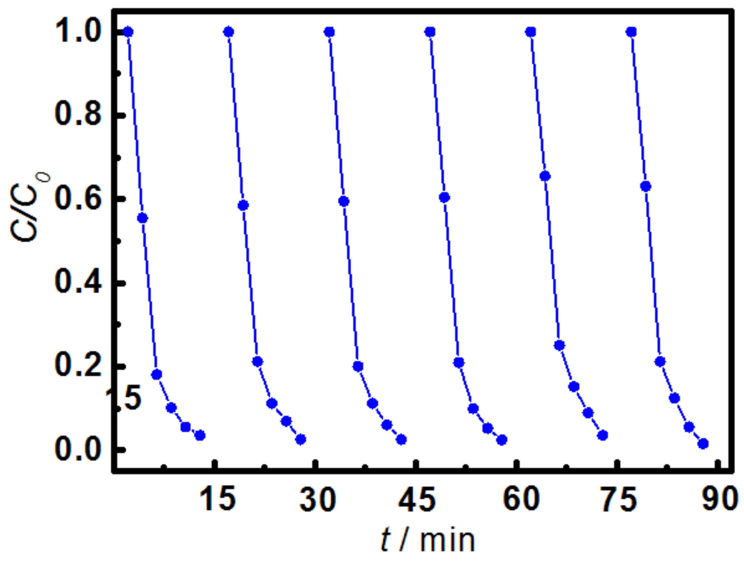

Figure S7. Recyclability of the photocatalytic decomposition of RhB with GWF.

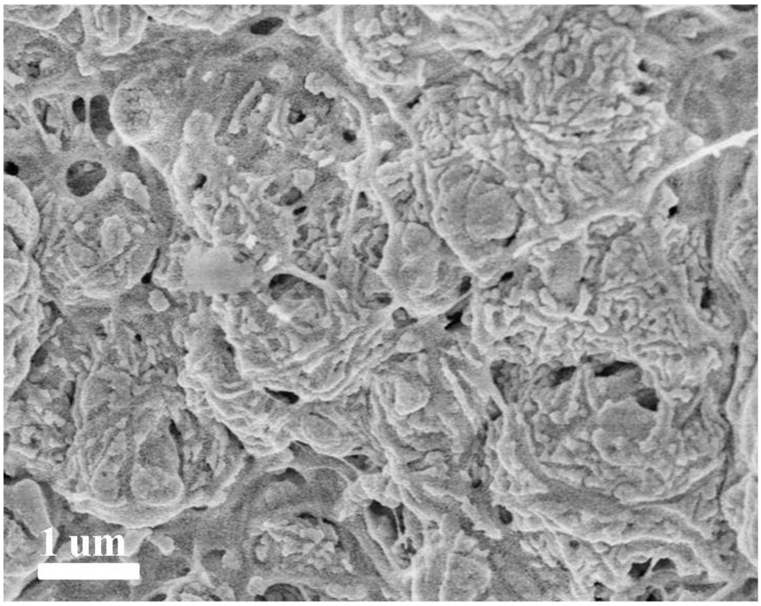

Figure S8. SEM images of GWF after six times photodegradation of RhB

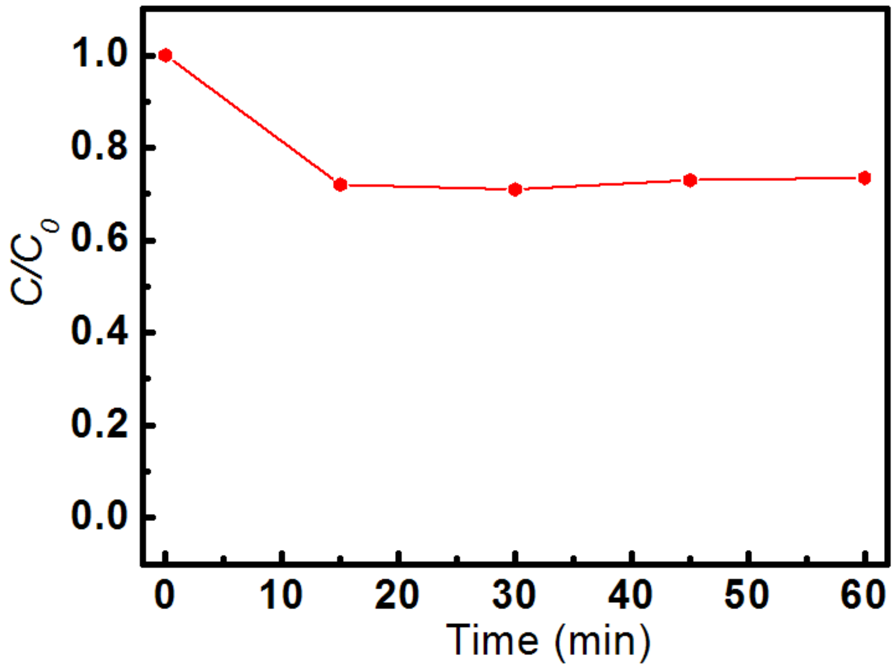

Figure S9. Photodegradation of RhB over GWF in dark.

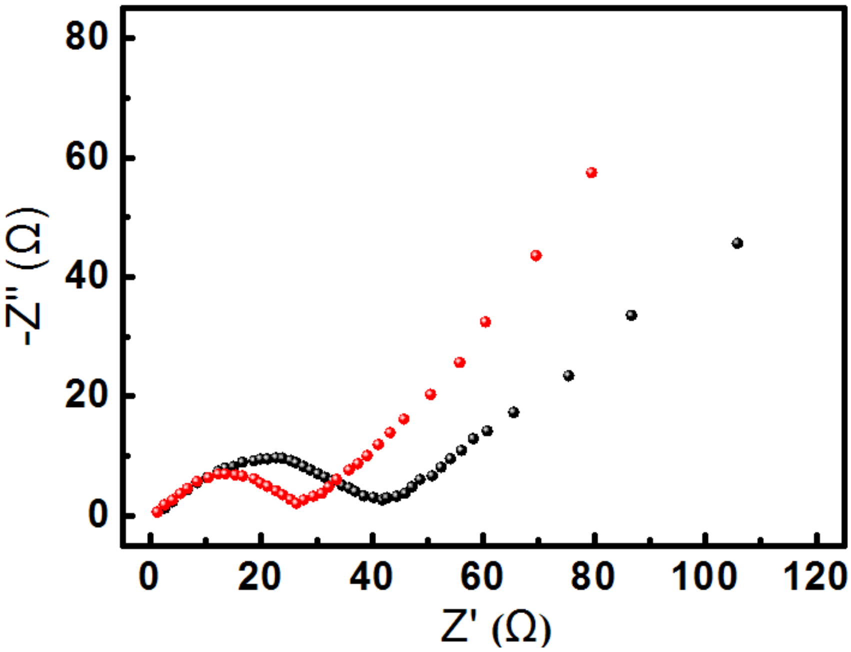

Figure S10. EIS changes of GWF (red dots) and WO_3 nanofibers
